# Editorial Department

**Published:** 1866-06

**Authors:** 


### Editorial Department.

Obscure Diseases of the Brain and Mind. By Forbes Winslow, M. D., D. C. L., Oxon. Second American from the third revised English edition. Philadelphia: Henry C. Lea, 1866.

The author, in placing this work before the medical profession, says that he "designs to briefly and clearly point out the more important, salient and characteristic symptoms that usually precede and accompany serious and often fatal attacks of the disease of the brain and disorders of the mind."

The obscurity of diseases of the brain renders them among the most difficult to treat successfully, and the physician is to guard with the greatest vigilance against, and if possible, to anticipate and prevent the stealthy approach of cerebral complications, especially as almost all affections of the brain, (according to the late Dr. Armstrong,) "though they appear to be sudden, will be found to be very slow. If the case, whether it occur in a congestive or in an excitive form, be traced backwards, you will find evidence of the patient having in the one case been in a most depressed state of mind, and in the other of having been in a most active state of mind, and in both cases it will appear that the stomach, liver, bowels, head and skin, were affected."

The masked affections of the mind receive much attention from the author; he says that "at the commencement of insanity the derangements of the intellect may be so slight and transient as to render the recognition as a formidable, impending malady, a task of great doubt and great difficulty, especially in the case of children. To the unskilled, untutored and untrained eye, the disease is, in its early stages, occasionally invisible. Even to the practical apprehension of the experienced physician, it is almost indiscernible, or at least of a dubious and uncertain character."

From the analysis of 2790 cases of insanity, contained in four American asylums, we learn that 33.73 per cent. were between the ages of twenty and thirty years, while in France and England the disposition to mental disease is far less at such an early period of life. The reason alleged for this is ascribed to the fact that the young men engage in business and politics at a far earlier age in the United States than is customary in the aforementioned countries. Almost all insane, the author with Esquirol claims, "exhibit, before their disease, some alterations in their functions, alterations which commenced many years previously, and even in infancy. The greater part had had convulsions, cephalagia, colics or cramps, constipation and menstrual irregularities. Several had been endowed with great activity in the mental faculties, and had been the sport of vehement, impetuous and angry passions. Others had been fantastical in their ideas, their affections, their passions; some had had an extravagant imagination, and been incapable of continuous study; others excessively obstinate, could not live, except in a very narrow circle of ideas and affections, whilst many, void of moral energy, had been timid, fearful, irresolute, indifferent to everything."

The responsibility of the medical witness when elucidating in courts of law, the phenomena of mental derangement, to enunciate principles, in advance of knowledge possessed by those who sometimes examine, and often severely, unjustly criticise him, cannot be sufficiently estimated, and we are exceedingly gratified in finding so much attention bestowed upon the subject of medical testimony in obscure cases of insanity. It is a too commonly received view that an alleged case of insanity must exhibit "all the usual stereotyped, artistic, poetic and melo-dramatic characters of madness, while the criminal lunatic almost invariably belongs to the class of quiet, cunning, subtle, *reasoning* madmen."

Many interesting and instructive examples of the diseases of the brain are given in this work, and it will prove to the professional practitioner a valuable acquisition, as it is writien in the most excellent style and contains as far as we are able to judge most of what is known on cases of the brain and mind.

---

*Transactions of the American Medical Association, vol. xvi. Philadelphia.*

This volume of the Transactions of the Association is a book consisting of 850 pages, and containing a large number of important reports on various subjects, some of which are very handsomely illustrated. Among those of particular interest we notice the paper of Dr. H. R. Storer, of Boston, "On the Causation, Course and Treatment of Insanity in Women;" his theory being, "that in women mental disease often, perhaps generally, depends upon functional or organic disturbance of the reproductive system." Several of the gentlemen on the committee with the doctor "admit that many cases of insanity in females are produced by uterine disturbance, but believe that too much importance may be attached to these disorders, and in our willingness to refer the mental symptoms to irritation, reflected from the uterus, we may lose sight of the cerebral disease." We think possibly the doctor is a little too sweeping in his opinions, and that while many cases of mental derangement in females can be traced to the reproductive system; yet a great many can also be referred to other causes. The report is well written, and contains some very interesting and instructive suggestions.

We also notice a report on the mechanical treatment of chronic inflammation of the joints of the lower extremities, with a description of some new apparatus for producing extension of the knee and ankle-joints, by Dr. Lewis A. Sayre, of New York. This report is practical and to the point in every respect. The description, use and application of the mechanical instruments, invented by the author, are clear and precisely explained, and cannot but lead to the most beneficial results if properly arranged.

The Committee on Medical Education, reported in favor of the colleges having a uniform and higher standard of education; they recommend that a larger number of "preparatory schools" be organized, and the student attend these instead of being in the office of a physician. The report says, "the medical education of youth ought to commence in a preparatory school, in which all the collateral branches of medicine should be taught." Preparatory medical schools are very good and should be supported, but still it depends upon many circumstances whether they are better, or even of as much value to the student as office practice, for the greater part of the more practical knowledge a young man possesses, when he graduates in medicine, is obtained in the office of his preceptor.

*West on the Diseases of Children.*

This well known author, upon the diseases of children, again appears in a fifth edition. Much of what he writes he has had opportunity to verify by repeated observation at the bed-side of the patients in the Children's Dispensary in Lambeth, and the Children's Hospital in Great Armond street. The present edition embodies the results of 1,200 recorded cases, and of nearly 400 *post mortem* examinations, collected from between 30,000 and 40,000 children who have been under his care in public or private practice.

This work has been long and favorably known to the profession, and this last edition will prove itself still more acceptable and valuable. The book comprises forty-four lectures, embracing nearly every subject pertaining to the diseases of children. The fullest instruction is given in the practical management of children, both medically and hygienically, thus rendering the book invaluable as a guide to the practitioner. It is for sale by medical booksellers generally, and should be in the hands of all physicians.

